# Gedunin Impacts Pancreatic Cancer Stem Cells Through the Sonic Hedgehog Signaling Pathway

**DOI:** 10.3390/ph19010019

**Published:** 2025-12-22

**Authors:** Karla Perez, Sheryl Rodriguez, Jose Barragan, Poornimadevi Narayanan, Alberto Ruiseco, Preetha Rajkumar, Nallely Ramirez, Victor Vasquez, Rajkumar Lakshmanaswamy, Ramadevi Subramani

**Affiliations:** 1 Center of Emphasis in Cancer Research, Department of Molecular and Translational Medicine, Paul L. Foster School of Medicine, Texas Tech University Health Sciences Center El Paso, El Paso, TX 79905, USA; 2 Francis Graduate School of Biomedical Sciences, Texas Tech University Health Sciences Center, El Paso, TX 79905, USA

**Keywords:** cancer stem cells, sonic hedgehog signaling, metastasis, pancreatic cancer, xenografts, gedunin

## Abstract

**Background/Objectives:** Pancreatic ductal adenocarcinoma (PDAC) is a highly aggressive cancer with a high rate of recurrence and a dismal prognosis. Studies have shown that pancreatic cancer stem cells (PCSCs) are a subpopulation that contributes to tumor progression, resistance to therapeutics, and metastasis, making them a key subpopulation to target for treatment. Gedunin (GD), a natural compound derived from *Azadirachta indica* (neem), has shown anticancer properties in pancreatic cancer cells, but its effects on PCSCs remains unclear. This study evaluated the effects of GD in pancreatic cancer stem cells, highlighting its impacts on tumor growth and progression and focusing on its impact on the sonic hedgehog (Shh) signaling pathway. **Methods:** Functional assays were performed to assess the effect of GD on the sphere-forming ability, colony formation, and self-renewal of PCSCs. Athymic mice xenograft models were utilized to evaluate the tumor suppression effect of GD in vivo. Furthermore, the anticancer effect of GD on PCSCs was assessed using both in vitro and in vivo limiting dilution assay. GD-induced changes in Shh signaling and key stem cell marker expressions in PCSCs were evaluated. **Results:** GD effectively inhibited tumor growth in xenograft models and reduced the percentage of PCSCs. GD was effective in decreasing PCSCs’ proliferative, self-renewal, and colony-forming capacity. GD decreased the protein expression levels of key Shh signaling markers Gli1 and Shh, stem cell markers SOX2, Nanog, and Oct4, metastasis-related proteins MMP-2, MMP-3, and MMP-9, and EMT markers Tgf1, Slug, Snail, and Twist in both PDAC cells and PCSCs. We demonstrated a significant decrease in the spheroid formation and self-renewal capacity of the (ALDH+) PCSC population following GD treatment in HPAC cells, indicating its potential antagonistic effects on PCSCs. GD was highly effective in reducing tumor volume, stemness, and metastasis in both early and late chemotherapy. In vivo limiting dilution assay using CD133+/LGR5+ PCSC xenografts demonstrated that GD reduces tumor growth, metastasis, and stemness associated with PCSCs by downregulating the expression of Shh and Gli1. GD treatment also reduced micrometastatic lesions in the lung, liver, and brain, as identified using H&E staining. **Conclusions:** The findings highlight GD’s potential as a promising therapeutic candidate for PDAC, with the ability to target both bulk tumor cells and PCSCs. By simultaneously suppressing tumor growth, stemness, and metastatic spread, GD may contribute to more effective treatment strategies and improved patient outcomes.

## 1. Introduction

Pancreatic cancer is a lethal malignancy, with global mortality rates closely matching its incidence rates. In the US, pancreatic cancer is the third leading cause of cancer-related death and is projected to become the second leading cause by 2030 [1]. Pancreatic ductal adenocarcinoma (PDAC) accounts for approximately 90% of all pancreatic cancer cases diagnosed [2]. PDAC is frequently diagnosed at advanced stages due to the absence of early symptoms. Current therapeutic strategies include surgery and chemotherapy [3]. The long-term survival rates remain dismal, and recurrence rates are high, largely owing to the development of chemoresistance [4,5].

Cancer stem cells (CSCs) are crucial to tumor progression, possess self-renewal capabilities, and are notoriously difficult to target and treat [6]. CSCs are also associated with increased resistance to chemotherapy and other therapeutic modalities [7]. These cells influence the tumor microenvironment and modify extracellular matrix regulation, further enhancing tumor progression [8]. CSCs contribute to the heterogeneity and plasticity of tumors [9]. Their dual capacity to promote both tumor progression and resistance to treatment underscores their importance as a pivotal target for therapeutic intervention. Studies have demonstrated the presence of a subpopulation of CSCs in PDAC, which plays a critical role in the growth and metastasis of PDAC [4]. These pancreatic CSCs (PCSCs) represent a potential therapeutic target for combating this highly aggressive cancer [4]. While PCSCs remain not fully characterized, they are believed to significantly influence both the chemoresistance and recurrence of PDAC. Specifically, gemcitabine treatment has been shown to enhance stemness, contributing to the development of chemoresistance in PDAC [10]. Therefore, targeting PCSCs is essential to overcome resistance and reduce recurrence, ultimately improving therapeutic outcomes for PDAC patients.

The sonic hedgehog (Shh) signaling pathway is a major regulator of PCSC self-renewal and maintenance of stemness [11,12]. Shh proteins secreted in this pathway initiate cellular responses such as proliferation and differentiation. Upon activation of Shh, the transmembrane receptor PTCH relieves the inhibition of SMO, another transmembrane protein, which in turn activates Gli1/2 proteins. These proteins dissociate from their repressor SUFU and translocate to the nucleus, where they regulate the transcription of downstream genes involved in tumor progression [13]. Gli1 is a pivotal component in Shh signaling, regulating stem cell properties, metastasis, the cell cycle, angiogenesis, epithelial-to-mesenchymal transition (EMT), and cell adhesion [14]. Various compounds, including natural products, have been shown to modulate SHH signaling pathways to inhibit tumor growth [15]. Preclinical studies have shown that GANT61, a direct Gli1 inhibitor, exhibits a potential antagonistic effect on PDAC development [16]. However, its clinical utility is limited by poor chemical stability in aqueous solutions, moderate activity requiring high doses in vitro, and the potential for acquired resistance through MAPK signaling, restricting its use to preclinical research [17]. Similarly, current clinical studies using SMO inhibitors (upstream pathway inhibitors such as sonidegib and vismodegib) face significant challenges due to SMO-independent activation of Gli1 and the development of drug resistance [18]. Our previous studies demonstrated that gedunin (GD) can significantly inhibit Gli1 and Shh expression, even in cells with high Shh levels, by targeting the Shh/Gli signaling pathway [19]. Therefore, novel strategies involving GD either alone or in combination with simultaneous inhibition of both canonical and non-canonical Shh pathways, along with targeting the PCSC population, may provide an effective approach to suppress PDAC progression.

Earlier, we demonstrated that bioactive molecules isolated from Azadirachta indica leaves possess potent anticancer effects [19,20,21,22]. Specifically, we reported that GD, a tetranortriterpenoid, effectively inhibits proliferation and impedes migration, invasion, epithelial-to-mesenchymal transition (EMT), and anchorage-independent growth in PDAC cells [19]. Moreover, GD induces apoptotic cell death in PDAC cell lines while exhibiting minimal toxicity toward normal pancreatic cells. Studies using recombinant Shh protein and GANT-61, a Gli1 inhibitor, indicate that GD exerts its anti-metastatic effects through inhibition of Shh signaling. Based on these findings, we hypothesize that GD suppresses PDAC progression by targeting pancreatic cancer stem cells (PCSCs) via the Shh/Gli1 signaling pathway, thereby reducing stemness, tumor growth, metastasis, and chemoresistance. We believe this study underscores the novelty and potential advantages of GD as a dual-targeting agent that modulates the Shh/Gli1 pathway and suppresses PCSC-driven tumor progression.

The aims of this study are to evaluate GD’s effects on PCSC self-renewal and stemness in vitro, assess tumor growth and metastasis in vivo, investigate modulation of Shh/Gli1 signaling, and explore GD’s potential as a therapeutic strategy to overcome chemoresistance and metastasis in PDAC. To the best of our knowledge, our study is the first to investigate the effects of gedunin on PCSCs specifically in relation to Shh signaling. Based on our in vitro and in vivo findings, we report that GD inhibits the growth and metastasis of PCSCs by modulating the Shh/Gli1 signaling pathway, demonstrating its promising anticancer effects in PDAC.

## 2. Results

### 2.1. Gedunin Inhibits Pancreatic Tumor Growth, Metastasis, and Sonic Hedgehog Signaling

**Figure 1:**
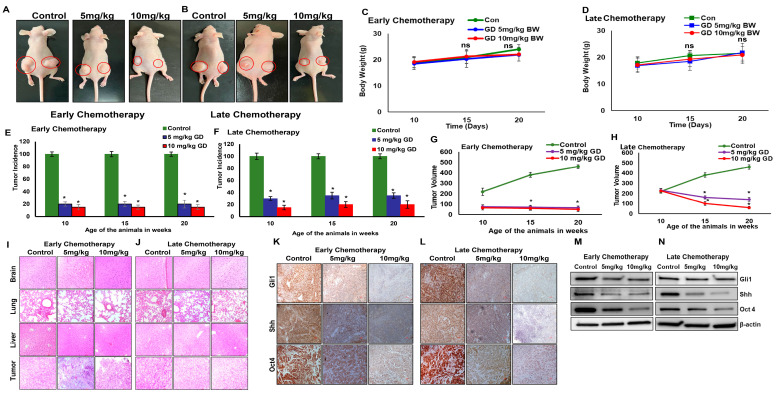
**Gedunin inhibits pancreatic tumor growth.** HPAC cells (1 × 10^6^ cells per flank) were injected subcutaneously into athymic nude mice and divided into two groups: early chemotherapy and late chemotherapy groups. (**A**) Early chemotherapy treatment groups: Control (Vehicle—DMSO), 5 mg/kg and 10 mg/kg body weight. (**B**) Late chemotherapy treatment groups: Control (Vehicle—DMSO), 5 mg/kg and 10 mg/kg body weight. Body weight of mice from early chemotherapy (**C**) and late chemotherapy (**D**) groups. Tumor incidence in GD-treated early chemotherapy (**E**) and late chemotherapy (**F**) groups. Tumor volume of early (**G**) and late chemotherapy (**H**) groups. H&E staining of brain, lung, liver, and tumor tissue of early (**I**) and late (**J**) chemotherapy groups. Immunohistochemistry analysis of Gli1, Shh, and Oct4 expression in tumors from early (**K**) and late (**L**) chemotherapy groups. Images were captured using Nikon ECLIPSE 50i Microscope at 20× magnification (**I**–**L**). Western blot analysis of Gli1, Shh, and Oct4 expression in early (**M**) and late (**N**) chemotherapy groups. Data are expressed as the mean ± SEM (* *p* < 0.05).

Athymic nude mice were implanted with pancreatic cancer cells and subsequently treated with either an early or late chemotherapy regimen, utilizing two dosages of GD (5 mg/kg and 10 mg/kg body weight). Significant reductions in tumor volume/size were observed in both early (Figure 1A) and late chemotherapy (Figure 1B) compared to control groups. Notably, GD treatment did not induce any substantial changes in body weight in early (Figure 1C) and late chemotherapy (Figure 1D) across all experimental groups, suggesting that the compound is well-tolerated. In the early chemotherapy regimen, GD-treated mice exhibited a lower tumor incidence (15–20%) over a period of 20 weeks, with minimal differences between the two dosages (Figure 1E). In the late chemotherapy regimen, too, both 5 mg/kg and 10 mg/kg body weight treatments were significantly effective in inhibiting tumor incidence by 70 and 80%, respectively, compared to controls (Figure 1F). Our data further demonstrated that in both early (Figure 1G) and late (Figure 1H) chemotherapy, both doses were highly effective in reducing tumor volume compared to the control groups. These observations clearly demonstrate the chemotherapeutic effects of GD in PDAC xenograft models.

Furthermore, the effect of GD on micrometastasis was evaluated using hematoxylin and eosin (H&E) staining. Our results revealed the distinct differences in micrometastasis in the lung, liver, and brain tissues of GD-treated animals at both 5 mg/kg and 10 mg/kg body weight doses when compared to control animals in the early (Figure 1I) and late chemotherapy groups (Figure 1J). Lung tissue from GD-treated mice exhibited thickened alveolar septa and cellular infiltration, both of which are indicative of metastatic activity. In the liver and brain, we observed fewer metastatic lesions in the GD-treated groups when compared to the control groups. Early (Figure 1I) and late (Figure 1J) chemotherapy with GD also resulted in alteration in tumor histology compared to the control. Tumor tissue from GD-treated mice demonstrated reduced cellular density and smaller nuclei, reflecting a significant reduction in cell proliferation. Overall, these findings confirm that GD treatment results in a significant reduction in tumor metastasis in PDAC. Furthermore, to validate the effect of GD on Shh signaling in PDAC, immunohistochemical analysis of tumor tissues was performed. The results demonstrated a notable reduction in the levels of Shh signaling pathway proteins, such as Shh and Gli1, as well as the stemness marker Oct4, in both the early (Figure 1K) and late chemotherapy groups (Figure 1L). This suppression of Gli1, Shh, and Oct4 was also confirmed by Western blot analyses in tumor tissue samples from both the early (Figure 1M) and late chemotherapy groups, compared to the control groups (Figure 1N). These above observations suggest that the GD treatment suppresses PDAC growth through targeting the Shh pathway.

### 2.2. Gedunin Decreases PCSC Population and Disrupts Stemness

**Figure 2:**
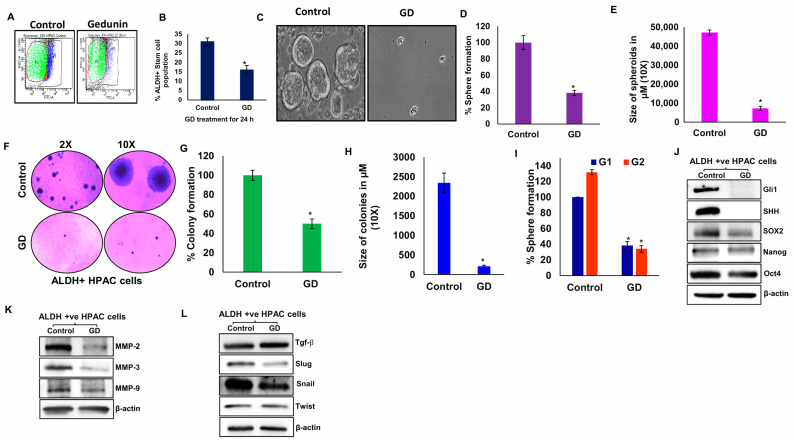
**Gedunin suppresses stemness, metastasis, and EMT in pancreatic cancer stem cells (PCSCs).** HPAC cells were treated with GD (25 µM) for 24 h, and ALDH+ population PCSCs were isolated. (**A**) Comparison of the ALDH+ PCSCs population in GD-treated HPAC cells. (**B**) Quantification of ALDH+ PCSC population in control and GD-treated groups. (**C**) Representative images of spheroids from control and GD-treated PCSCs and (**D**) quantification of spheroid-forming capacity in GD-treated and control groups. (**E**) Spheroid size in GD-treated and control groups. (**F**) Colony formation images at 2× and 10× magnification. (**G**) Quantification of colonies. (**H**) Size of colonies in GD-treated and control groups. (**I**) Self-renewal capacity assessed by secondary spheroid formation assay. (**J**–**L**) Western blot analysis of Gli1, SHH, SOX2, Nanog, and Oct4 (**J**); MMP2, MMP3, and MMP9 (**K**); and Tgfβ, Slug, Snail, and Twist (**L**). β-actin was used as an internal control for all Western blots. Data are expressed as means ± SEM (* *p* < 0.05).

Since there was a notable reduction in Oct4 in GD-treated xenografts, we explored the effect of GD on PDAC stemness. For this, we examined the effect of GD on PCSCs, a subpopulation critical for driving tumor progression and resistance to therapy. After 24 h of GD treatment, ALDH+/PCSCs were isolated from HPAC cells. There was a significant reduction in ALDH+/PCSCs from 31% in controls to 16% upon GD treatment (Figure 2A,B). This observation confirms that GD treatment impacts the percentage of PCSCs.

To validate the anti-stemness property of GD, we further examined the spheroid-forming ability of the PCSCs that were isolated from GD-treated HPAC cells. Our data demonstrated a dramatic reduction in both sphere number (~60%) (Figure 2C,D) and size (~40%) (Figure 2E) following GD treatment compared to the control. Furthermore, we examined the colony formation abilities in GD-treated ALDH+/PCSCs. The size and number of colonies were measured. We observed a ~50% decrease in colony number and a substantial reduction in colony size (Figure 2F–H). Additionally, we analyzed the second-generation sphere formation ability, and our observations revealed a significant impairment in the self-renewal capacity of GD-treated cells (Figure 2I). These observations clearly showed that the GD has an inhibitory effect on colony-forming and self-renewal capabilities of PCSCs.

To further understand the anti-stemness effect of GD at the molecular level, we examined the expression of Shh pathway proteins (Gli1 and Shh) and stem cell markers (SOX2, Nanog, Oct4) using Western blot analysis in ALDH+/PCSCs. Our results showed that GD treatment led to the downregulation of key PCSC markers and Shh signaling components (Figure 2J). Furthermore, GD treatment suppressed the expression of matrix metalloproteinases (MMP-2, MMP-3, and MMP-9) (Figure 2K) and EMT markers (Tgf-β, Snail, Slug, Twist) (Figure 2L), which play pivotal roles in PCSC-mediated metastasis. Collectively, these findings demonstrate that GD effectively targets PCSCs by impairing shh signaling, stemness, EMT and tumor microenvironment.

### 2.3. Gedunin Selectively Targets CD133^+^ and LGR5^+^ Pancreatic Cancer Stem Cells

**Figure 3:**
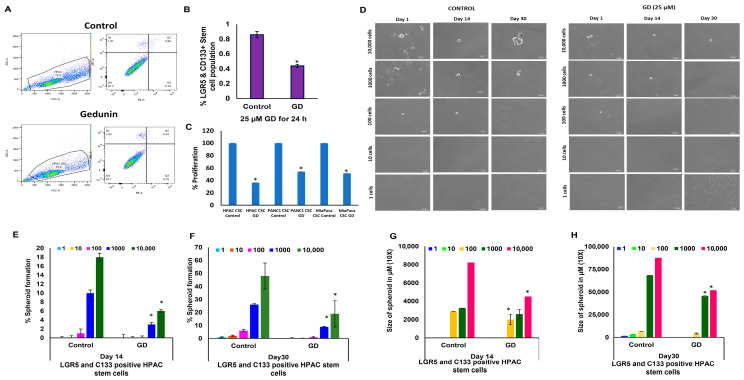
**CD133^+^ and LGR5^+^ PCSCs are altered by gedunin.** HPAC cells were treated with GD (25 µM) for 24 h, and CD133^+^ and LGR5^+^ PCSC populations were isolated. (**A**) Flow cytometric analysis of the effect of GD on CD133^+^ and LGR5^+^ PCSC populations. (**B**) Quantification of LGR5^+^ and CD133^+^ PCSCs in the control and GD-treated group. (**C**) Effect of GD on HPAC, PANC-1, and MIA PaCa-2 CD133^+^ and LGR5^+^ PCSC proliferation. (**D**) In vitro limiting dilution assay (LDA) at 1, 14, and 30 days post GD treatment, imaged using a light microscope at 20× magnification. (**E**,**F**) The percentage of spheroid formation was quantified after 14 (**E**) and 30 (**F**) days of GD treatment. (**G**,**H**) The average size of the spheroids was measured at 14 (**G**) and 30 (**H**) days following GD treatment. Data are expressed as means ± SEM (* *p* < 0.05).

To further investigate the effects of GD on distinct PCSC subpopulations, we focused on CD133^+^ and LGR5^+^ cells, which are specific markers for PCSCs. Notably, GD treatment led to a significant reduction in the expression of both LGR5^+^ and CD133^+^ in ALDH-sorted cells (Figure S1). The purity of the sorted cell populations was confirmed, achieving 99.6% for CD133^+^ and 99.8% for LGR5^+^ cells (Figure S2). Flow cytometric analysis revealed a substantial decrease in the CD133^+^ and LGR5^+^ cell populations following GD treatment, from 0.86% in control tumors to 0.44% in GD-treated tumors (Figure 3A,B).

To analyze the effect of GD on the proliferation rate of the PCSCs, we sorted (LGR^+^/CD133^+^) PCSCs from PDAC cell lines such as HPAC, PANC1, and MIA PaCa-2, and the percentage of proliferation was determined using the MTS assay. PCSCs were isolated from the PDAC cell line after GD treatment for 24 h. GD treatment resulted in a marked reduction in PCSC proliferation across all three cell lines when compared to the control group (Figure 3C). Additionally, we examined the self-renewal capability using a limited dilution assay (LDA) for 30 days. The results showed a significant reduction in the size and number of spheroids in the PCSCs from GD-treated HPAC cells (Figure 3D). Specifically, PCSCs from the control group were capable of forming spheroids at as few as 1000 cells/well, while PCSCs from GD-treated HPAC cells exhibited impaired spheroid formation, even at higher cell concentrations. By day 30, the spheroids of PCSCs from GD-treated HPAC cells were fewer in number and exhibited reduced density compared to controls, indicating compromised proliferation and aggregation capacity. The sphere formation in PCSCs of GD-treated HPAC cells displayed a significantly lower percentage across all groups compared to controls at both 14 (Figure 3E) and 30 days (Figure 3F). Similarly, GD treatment led to a marked reduction in spheroid size compared to controls at both 14 (Figure 3G). and 30 days (Figure 3H). Together, these findings confirm that GD targets CD133^+^ and LGR5^+^ PCSC populations, impairing their self-renewal, proliferation, and spheroid formation capacity, underscoring its potential as a therapeutic agent for targeting PCSCs in PDAC.

### 2.4. Gedunin Modulates Gene Expression and Inhibits PCSC Tumorigenicity

**Figure 4:**
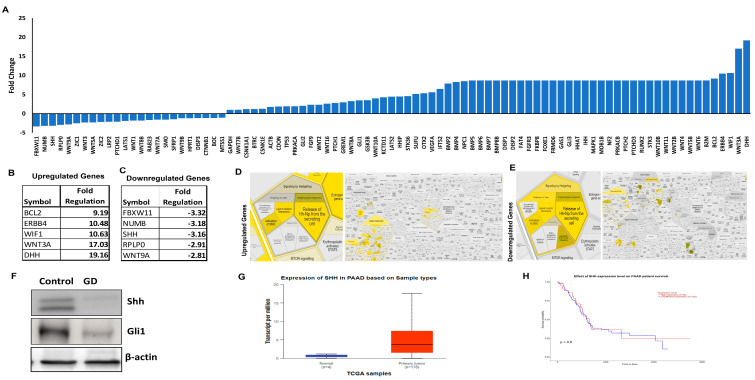
**Gedunin targets the sonic hedgehog pathway (Shh) in PCSCs.** HPAC cells were treated with GD (25 µM) for 24 h, and CD133^+^ and LGR5^+^ PCSC populations were isolated and cultured for 21 days. (**A**) Waterfall plot illustrating GD-induced differential gene expression in human Shh signaling pathway. (**B**,**C**) List of the top 5 upregulated (**B**) and downregulated (**C**) genes in response to GD. (**D**,**E**) Reactome pathway analysis of upregulated (**D**) and downregulated (**E**) genes highlighting key signaling processes affected by GD treatments in PCSC. (**F**) Western blot analysis of Shh and Gli1 expression in GD-treated PCSCs. Data were normalized with β-actin expression. (**G**) Expression levels of Shh in tumor samples from the TCGA dataset. (**H**) Kaplan–Meier survival analysis demonstrating the effect of Shh expression on patient survival outcomes. Data are expressed as means ± SEM.

To further investigate the effect of GD on shh signaling, we performed RT^2^ profiler PCR array for genes in the human sonic hedgehog pathway (Shh). Differential gene expression revealed that GD substantially up- and downregulated key genes involved in the shh signaling pathway (Figure 4A). BCl2, ERBB4, WIF1, WNT3A, and DHH were among the most significantly upregulated genes (Figure 4B). The most significantly downregulated genes were FBXW11, NUMB, SHH, RPLP0, and WNT9A (Figure 4C). Notably, the suppression of SHH and WNT9A aligns with GD’s impact on the Shh signaling pathway, both of which are essential for maintaining PCSC stemness and tumor progression [19]. Additionally, the downregulation of FBXW11 and NUMB suggests potential alterations in the Notch and Wnt signaling pathways, further compromising stemness.

To investigate the interaction between GD and altered genes of the Shh pathway, we have performed the Reactome analysis. Reactome analysis of the top differentially expressed genes (Figure 4D,E) provided insights into the specific loci within the Shh pathway affected by GD. Both upregulated and downregulated genes were associated with key events such as the activation of SMO, ligand–receptor interactions, and the release of Hh-Np from secreting cells. Intriguingly, the downregulated genes also highlighted the activation of the proteasomal degradation of Gli1, a crucial event in suppressing tumor proliferation. To validate these findings, Western blot analysis was conducted, confirming a downregulation of Shh and Gli1 in the GD-treated group compared to the control (Figure 4F). Notably, SHH, which is highly expressed in patient tumors (Figure 4G) and correlates with poor patient survival (Figure 4H), was significantly reduced in GD-treated PCSCs. Overall, these results indicate that GD inhibits the stemness of PDAC by altering Shh signaling proteins in PCSCs.

### 2.5. Gedunin Decreases PCSC-Associated Tumor Progression in Xenograft Models

**Figure 5:**
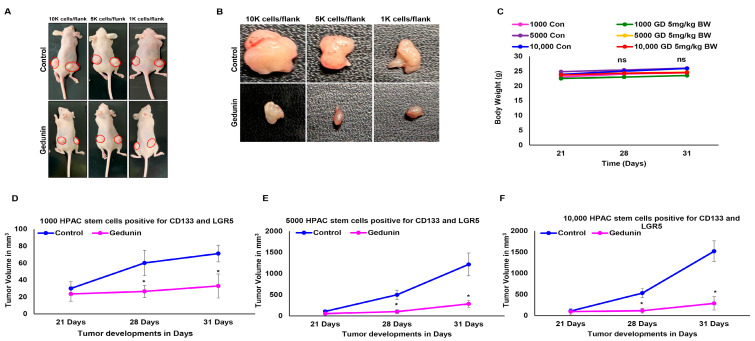
**Gedunin suppresses PCSC tumor growth in xenograft models.** A limiting dilution assay was performed using PCSCs that were sorted from the HPAC cell line and injected subcutaneously into athymic nude mice with 10 k, 5 k, or 1 k cells per flank. (**A**) Representative images of control and GD-treated PCSC xenograft tumors in athymic mice injected with 10 k, 5 k, or 1 k PCSCs per flank. (**B**) Excised tumors from 10 k, 5 k, and 1 k PCSC-transplanted groups. (**C**) Comparison of body weight and (**D**–**F**) comparison of tumor volume over time in control and GD-treated groups with 1 k (**D**), 5 k (**E**), and 10 k (**F**) PCSC xenografts. Data are expressed as the mean ± SEM (* *p* < 0.05).

To investigate the effect of GD on PCSC in vivo, we performed an LDA using the (LGR^+^/CD133^+^) PCSCs from the HPAC cell line and injected them into athymic nude mice subcutaneously at 1000, 5000, or 10,000 cells per flank. The tumor growth and volume were monitored every week. After the tumor size had reached 100 mm^3^, GD (5 mg/kg of body weight) was injected intraperitoneally twice a week. We observed that tumor development was significantly inhibited in the GD-treated groups (Figure 5A,B). Tumor volume was remarkably reduced. No significant change in body weight was observed between the control and GD-treated groups (Figure 5C). Tumor volume was remarkably reduced in all the PCSC limiting dilution groups, namely 1000 (Figure 5D), 5000 (Figure 5E), and 10,000 (Figure 5F), compared to the respective controls. These important observations demonstrate the inhibitory effect of GD on PCSCs’ tumor-forming capacity.

**Figure 6:**
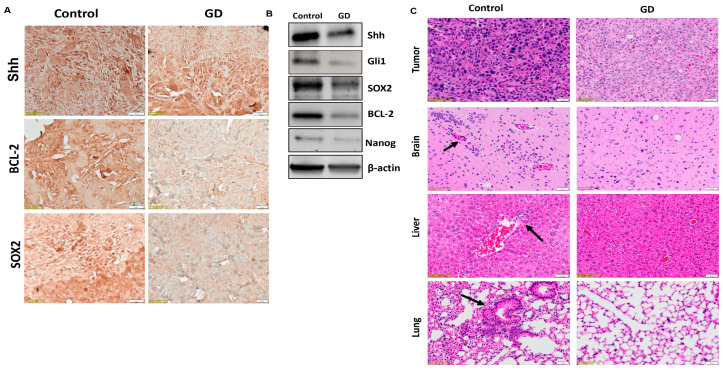
**Gedunin targets Shh/Gli1 signaling in PCSC-induced tumors in xenograft models.** (**A**) Immunohistochemistry analysis of Shh, BCL-2, and SOX2 expression in control and GD-treated tumors. (**B**) Western blot analysis of Shh, Gli1, SOX2, BCL-2, and Nanog expression in control and GD-treated PCSC xenografts. Data were normalized with β-actin expression. (**C**) H&E staining of tumor, brain, liver, and lung tissue in control and GD-treated groups. All data are from the 10 K xenograft groups. Images were captured using an Olympus APEXVIEW APX100 using 10× magnification.

Additionally, we investigated the expression of Shh, Bcl2 (anti-apoptotic protein), and SOX2 (stemness marker) proteins in the PCSC xenograft tumors. We found that PCSC tumors of the GD-treated group showed a significant reduction in the expression of Shh, Bcl-2, and SOX2 compared to the controls (Figure 6A). To confirm the above findings, we performed Western blot analysis of Shh, Bcl2, and SOX2 proteins. The results showed a consistent downregulation of SHH, Bcl-2, and SOX2 (Figure 6B) in the GD-treated group, validating the immunohistochemical findings. Similar trends in SHH expression following GD treatment were observed in both parental HPAC xenograft tumors (Figure S3) and 5K PCSC xenografts (Figure S4). Additionally, the stemness marker Nanog was downregulated in GD-treated tumors, while Gli1 showed a modest decrease. To confirm the anti-metastatic capability of GD, we analyzed brain, liver, lung, and tumor tissues using H&E staining. The results showed that metastatic lesions in liver, lung, and brain tissue were more prevalent in the control group compared to the GD-treated groups (Figure 6C), suggesting that GD effectively impedes PDAC growth and metastasis driven by PCSCs. These results reinforce GD’s ability to disrupt the capacity of PCSCs to promote PDAC progression through the modulation of the Shh signaling pathway.

## 3. Discussion

Clinical evidence indicates that PDAC frequently recurs within six months following surgical resection [23]. Given the substantial role of PCSCs in tumor progression, recurrence, and metastasis, recent therapeutic strategies have increasingly focused on targeting these cells [24]. Our previous in vitro and in vivo work demonstrated that GD inhibits PDAC growth and metastasis through modulation of the Shh/Gli1 signaling axis [19]. Building upon these findings, the current study provides first convincing evidence that GD exhibits potent anti-tumor activity against both bulk PDAC cells and the highly tumorigenic PCSC population. The observed reduction in tumor size following GD treatment suggests its efficacy in targeting the bulk tumor mass, while its ability to downregulate key markers and impair self-renewal capacity reinforces GD’s potential in targeting the PCSCs. GD administration resulted in a dose-dependent reduction in tumor volume in bulk PDAC cells. It significantly downregulated OCT4, a stem cell marker in GD-treated xenograft tumors of HPAC cells. This reduction in Oct4 expression prompted further investigation into the effects of GD on PCSCs, which are key drivers of tumor recurrence, metastasis, and resistance to standard therapies in PDAC [4].

PCSCs are critically involved in progression due to their self-renewal capacity and ability to evade conventional treatments [6,7]. Wu et al. reported that ALDH family proteins are associated with poor prognosis in PDAC patients [25]. Our results convincingly demonstrate that GD effectively reduces the activity of ALDH^+^ PCSCs, as evidenced by a marked reduction in colony formation and tumor growth. Previous studies have shown that the inability to form colonies in culture contributes to a decreased likelihood of metastasis and may also increase cancer cell sensitivity to chemotherapy [26]. We observed a significant reduction in the percentage of CD133^+^ and LGR5^+^ PCSCs following GD treatment, highlighting GD’s ability to target this critical population of cells associated with chemoresistance and metastasis. GD demonstrated a remarkable reduction in the aggressiveness of these PCSCs both in vitro and in vivo, emphasizing its potential to counteract the tumor-initiating and promoting capacity of PCSCs. These findings are significant, as PCSCs contribute to tumor heterogeneity and resistance, making them an essential target for achieving durable responses in PDAC treatment [5,9]. A key discovery in this study is the modulation of the Shh signaling pathway by GD in both bulk cancer cells and PCSCs. The Shh pathway plays a pivotal role in regulating PCSC self-renewal, tumor growth, and metastasis, and its dysregulation is strongly associated with poor prognosis in PDAC patients [8]. Our in vivo LDA results demonstrate that GD disrupts PCSC tumorigenicity, with tumors derived from GD-treated PCSCs showing a significant reduction in tumor volume compared to tumors from untreated PCSCs. GD effectively downregulated critical components of the Shh pathway, including SHH itself, and appears to regulate the downstream target BCL-2, which is involved in apoptosis regulation [27]. GD, a known Hsp90 inhibitor, is considered a crucial therapeutic agent in cancer treatment [28,29]. It has been shown to induce apoptosis in lung cancer cells by disrupting the Hsp90–Beclin-1–Bcl-2 interaction, leading to the downregulation of autophagy [30]. While previous studies in pancreatic cancer cells have reported a significant role of Gli1, a transcription factor central to Shh-mediated gene expression in promoting cell survival [31,32], our data specifically demonstrate a reduction in Gli1 expression within PCSCs. Moreover, GD treatment also affected the expression of Oct4, SOX2, and Nanog, key regulators of stemness. These findings suggest that GD may interfere with critical stemness-related pathways, resulting in reduced aggressiveness of PDAC. GD has been identified as a novel microtubule-inhibiting drug candidate [33]. Cancer stem cells (CSCs) rely on intact microtubule networks for essential functions, including the maintenance of asymmetric cell division and the regulation of intracellular trafficking required for stemness. Consequently, microtubule-targeting drugs can reduce self-renewal capacity, impair sphere-forming ability, and downregulate key stemness-related transcription factors. Cancer types driven by sonic hedgehog (Shh) signaling, such as PDAC, may be particularly sensitive to microtubule-interfering compounds, as primary cilia and microtubule structures play a central role in Shh pathway activation [34,35]. Our data suggest that GD’s inhibitory effects on PCSCs may involve modulation of the Shh/Gli1 signaling axis in conjunction with microtubule inhibition. This dual mechanism represents an attractive therapeutic strategy for targeting PCSCs, which are often highly resistant to standard chemotherapy.

Enhanced tumor formation and metastasis are closely associated with dysregulation of several biological processes involving cancer stem cells and their specialized microenvironment [36]. GD’s ability to suppress metastasis further aligns with its inhibitory effects on PCSCs. We have shown earlier that GD reduces EMT, a critical process for metastasis often driven by CSCs [19]. Further, it has also been demonstrated that inhibition of shh remodeled stroma and increased the anticancer effect of pancreatic cancer therapeutics [37]. Our findings extend this observation by demonstrating that GD’s inhibition of the Shh pathway in PCSCs contributes to the observed reduction in EMT, colony formation, and metastasis of PDAC.

While our findings are compelling, there are certain limitations that must be acknowledged. Using orthotopic and patient-derived xenograft (PDX) models would better recapitulate the PDAC tumor microenvironment, and establishing the direct molecular interaction between GD and Shh pathway would enhance our understanding of the molecular mechanism by which GD induces its anticancer effect. Our future research will explore combination therapies integrating GD with standard chemotherapeutics or Shh pathway inhibitors to enhance treatment efficacy and overcome resistance.

## 4. Materials and Methods

### 4.1. Cell Lines

The pancreatic ductal adenocarcinoma (PDAC) cell lines HPAC, Mia-Paca2, and PANC-1 were obtained from the American Type Culture Collection (ATCC) and cultured at 37 °C in a humidified incubator with 5% CO_2_.

### 4.2. Apoptosis Analysis and Flow Cytometry

Apoptosis was assessed using the Annexin V-FITC Apoptosis Detection Kit (BD Biosciences, San Diego, CA, USA; #556547), following the manufacturer’s instructions. Briefly, cells were seeded at 5 × 10^5^ cells/well in 6-well plates and treated with 20 µM GD for 24 h. After treatment, cells were washed twice with cold PBS and resuspended in 1× binding buffer. Annexin-V was added, followed by a 15 min incubation at room temperature in the dark. Cells were washed again, incubated with 2 µL of propidium iodide (PI) for an additional 15 min, and analyzed using the FACS Accuri C6 flow cytometer (BD Biosciences, San Jose, CA, USA).

### 4.3. Cell Sorting

Pancreatic cancer stem cells (PCSCs) were isolated using the ALDEFLUOR ALDH kit (StemCell Technologies, Vancouver, BC, Canada #01700) or with CD133 (Biolegend, San Diego, CA, USA #373804) and LGR5 (Biolegend #397906) as markers. Cells were seeded at 5 × 10^5^ cells per well in a 6-well plate and treated with 25 µM GD for 24 h before sorting. Untreated cells served as controls. Cells were trypsinized and pelleted. Magnetic bead-based cell sorting was performed using “The Big Easy” EasySep Magnet (StemCell Technologies #18001), as per the manufacturer’s protocol. In brief, the cell pellet was prepared for sorting by incubating with FcR blocker and CD 133 APC-conjugated or LGR5 PE-conjugated antibodies. After 15 min at room temperature, the selection cocktail was added and incubated for an additional 15 min. Then, Rapid Spheres were added, and cells were incubated for another 10 min. The sorted LGR5+/CD133+ PCSCs were then used for further analysis.

### 4.4. Sphere Formation and Self-Renewal Assay

The sphere formation assay was performed as previously described [38]. Briefly, PCSCs isolated from control and GD-treated (25 µM) cells were plated as a single-cell suspension with the use of a 23 G needle. They were plated in ultra-low attachment 6-well plates with sphere formation media containing B27 supplement and single quots (hydrocortisone, insulin, beta mercaptoethanol, EGF, and gentamycin) in phenol red-free DMEM/F12 media (GIBCO, Waltham, MA, USA). The cell density for this assay was optimized to 1000 cells/cm^2^. The cells were not disturbed for 5 days before any media change. After 7 days, any sphere larger than 50 µm was counted under a phase contrast light microscope and used for further analysis to find the effect of GD on self-renewal. Stem cells were isolated using ALDH as a marker. For the self-renewal assay, the 1st-generation spheres from the above-mentioned groups were dissociated using Accutase, (Innovative Cell Technologies, Inc., San Diego, CA, USA, counted, and re-plated in the same media as single cells with a plating density of 5000 cells/well. The cells were incubated in a 37 °C, 5% CO_2_ humidified incubator for 7 days. From day two onwards, we monitored each well using a phase contrast microscope (Nikon Eclipse TS100; Nikon, Melville, NY, USA) and photographed the cells as necessary. We used 5 replicates for sensitive readouts. The same process was followed for the subsequent generations.

### 4.5. Colony Formation

ALDH-positive cells isolated from control and GD-treated groups were seeded at 1.5 × 10^5^ cells per 60 mm Petri dish containing a bottom layer of 1% agar (Becton Dickinson, San Jose, CA, USA #DF001-17-0) and a top layer of 0.7% agarose (Agarose Ultrapure, Invitrogen, Carlsbad, CA, USA #16500-100). Cells were fed twice weekly and cultured for up to 45 days. Colonies were stained with 0.2% crystal violet for 1 h and imaged using a Nikon SMZ 1500 microscope (Nikon, Melville, NY, USA) at magnifications of 2× and 10×.

### 4.6. Immunoblotting

Total protein from both in vitro PDAC cell cultures and xenograft tumor tissues were extracted and subjected to SDS-PAGE, followed by transfer onto PVDF membranes. Membranes were blocked with 5% bovine serum albumin (BSA) and incubated overnight at 4 °C with primary antibodies. The proteins assessed include Gli1 (Cell Signaling Technology, Danvers, MA, USA #3538P), Shh (Cell Signaling Technology, #2207P), Nanog (Cell Signaling Technology, #4903S), Sox2 (Cell Signaling Technology, #3579S), Oct4 (Cell Signaling Technology, #2750S), Slug (Cell Signaling Technology, #9585S), Snail (Cell Signaling Technology, #3879S), BCL-2 (Cell Signaling Technology, #3498S), MMP-3 (Abcam, Cambridge, MA, USA #ab52915), Twist (Abcam, #ab50887), MMP-2 (Santa Cruz, Dallas, TX, USA #SC-53630), MMP-9 (Santa Cruz, #SC-21733), TGF-β (Thermo Fisher, Waltham, MA, USA #MA5-16940), and ß-actin (Sigma-Aldrich, St. Louis, MO, USA #A1978). The appropriate secondary antibodies were used. The blots were developed using enhanced chemiluminescence.

### 4.7. Limiting Dilution Assay (LDA)

In vitro LDA was conducted using HPAC stem cells in the presence of 25 μM GD to assess the impact on sphere-forming capacity. Cells were seeded in ultra-low attachment plates at various densities ranging from 10,000 to 1 cell per well and observed for up to 30 days. Sphere formation was monitored by measuring and counting the spheres at days 1, 14, and 30 under a light microscope at 20× magnification. Representative images were captured to compare the GD-treated versus control groups.

For in vivo studies, PCSCs (10,000, 5000, and 1000 cells/100 µL) were injected subcutaneously into athymic nude mice to evaluate GD’s effect on tumor development. Each experimental group consisted of six mice with balanced sex distribution. Tumor growth was monitored weekly, and tumor volume was calculated using the formula 4/3π × r_1_^2^ × r_2_, where r_1_ is the minor radius and r_2_ is the major radius. Body weight was also measured weekly post-injection. All animal experiments were approved (18 June 2025) by the Texas Tech University Health Sciences Center El Paso Institutional Animal Care and Use Committee.

### 4.8. Proliferation Assay

The effect of GD on cell proliferation was assessed using the Cell Titer 96^®^ Aqueous One Solution Cell Proliferation Assay (Promega, Madison, WI, USA #G358B) according to the manufacturer’s protocol. In brief, 6000 PCSCs were seeded in a 96-well plate overnight, and cells were treated with GD at 25 µM for 24 h followed by 20 µL of MTS reagent being added to each well containing 100 µL of media. The plate was incubated for 4 h at 37 °C in a 5% CO_2_ incubator. Absorbance was measured at 490 nm using a Clariostar reader, BMG LABTECH, Cary, NC, USA. Proliferation of each cell line was calculated in comparison to the control group.

### 4.9. RT^2^ Profiler™ PCR Array Human Sonic Hedgehog Signaling Pathway

HPAC stem cells were cultured for 21 days at 37 °C with 5% CO_2_. Control and GD-treated cell pellets were collected and RNA was extracted using the TRIzol method. RNA quantity and quality were assessed using a Nanodrop 2000 spectrophotometer, Waltham, MA, USA. RNA (2 µg) was converted to cDNA using the Revert Aid First Strand cDNA Synthesis Kit (Thermo Scientific, Agawam, MA, USA). The cDNA was mixed with RT^2^ SYBR^®^ Green Master Mix (Qiagen, Germantown, MD, USA) and loaded onto an RT^2^ Profiler™ PCR Array for the Human Sonic Hedgehog Signaling Pathway. PCR amplification was carried out using a Roche LightCycler system according to the manufacturer’s instructions.

### 4.10. Pathway and Gene Expression Analysis

Differential gene expression data were analyzed using Reactome (reactome.org) to identify affected biological pathways. The top upregulated and downregulated genes were assessed for their involvement in relevant signaling pathways. Additionally, SHH expression was evaluated in tumor samples using UALCAN (ualcan.path.uab.edu) to generate Kaplan–Meier survival plots.

### 4.11. Xenograft Model

All animal experiments were approved (18 June 2025) by the Texas Tech University Health Sciences Center El Paso Institutional Animal Care and Use Committee. HPAC cells (1.0 × 10^6^ cells per flank) were injected subcutaneously into the right and left flanks of seven-week-old athymic nude mice. Experimental groups included a control group (0.1% DMSO) and GD-treated groups (5 mg/kg and 10 mg/kg body weight). GD treatment was administered intraperitonially twice a week, either two weeks post-transplantation (early chemotherapy) or after the appearance of a palpable tumor (regular chemotherapy) for 4 weeks. Tumor volume and body weight were monitored weekly. Tumor volume was compared between the control and GD treatment groups using the formula 4/3π × r_1_^2^ × r_2_, where r_1_ is the minor radius and r_2_ is the major radius. The mice were euthanized after 4 weeks of GD treatment by CO_2_ overdose at a flow rate of 50% L/minute to collect tumors, as well as tissues from various organs (brain, lung, and liver) for further analysis. A portion of each of the tissues was fixed in 10% formalin for histopathological analysis and immunohistochemistry. The remainder of the tissue sample was flash-frozen in liquid nitrogen to be used for molecular analysis.

### 4.12. Hematoxylin and Eosin (H&E) Staining

Formalin-fixed paraffin-embedded tissue sections were deparaffinized, rehydrated, and stained with hematoxylin and eosin (H&E). Micrometastatic lesions in the brain, liver, and lung tissues from both control and GD-treated mice were evaluated under a microscope. Images were captured using Nikon ECLIPSE 50i Microscope (Nikon, Melville, NY, USA) at 20× magnification.

### 4.13. Immunohistochemistry (IHC)

Formalin-fixed, paraffin-embedded tumors were deparaffinized using xylene and rehydrated via decreasing ratios of ethanol baths. After antigen retrieval, tissue sections were blocked and incubated with antibodies for SHH, SOX2, and BCL-2. Tissue sections were stained with DAB chromogen and counterstained with hematoxylin, followed by dehydration through increasing ethanol dilutions ending in a xylene bath. Then, mounting media (Surgipath Medical Industries, Richmond, IL, USA) was added, and the coverslip was mounted onto the slide. Images were captured using an Olympus APEXVIEW APX100 (Evident Corporation, PA, USA).

### 4.14. Statistical Analysis

Data analysis was performed using Graphpad Prism version 10.4. Data are expressed as means ± standard deviations (SDs). Student’s ‘*t*’ test was used to compare between two groups and ANOVA was used to compare multiple groups. *p* < 0.05 was considered statistically significant. All in vitro studies and other technical procedures were repeated at least 3 times, and all in vivo experiments had 6 animals per group.

## 5. Conclusions

To the best of our knowledge, our study is the first to investigate the effects of gedunin on pancreatic cancer stem cells (PCSCs) specifically in relation to sonic hedgehog (Shh) signaling. While in our previous studies GD was shown to exert anticancer activities in pancreatic cancer cell lines through induction of apoptosis, suppression of migration/invasion, and downregulation of Shh/Gli pathway components (e.g., Shh, PTCH1/2, SUFU, GLI1), those findings were obtained exclusively in bulk pancreatic cancer cell populations [19]. The effects of GD on PCSCs have not been studied. This gap is significant because extensive research demonstrates that Shh/Hedgehog signaling is a critical regulator of PCSC maintenance, driving self-renewal, tumor initiation, metastasis, and therapeutic resistance.

By directly testing the effects of GD on PCSCs and assessing Shh/Gli signaling in this context, our study fills an important and previously unaddressed gap in the literature. This adds mechanistic depth to earlier GD reports and provides new insights into its potential to target the PCSC population that drives pancreatic cancer aggressiveness, recurrence, and therapy resistance. In conclusion, our data demonstrates that GD significantly inhibits PDAC growth, stemness, and metastasis by altering the Shh signaling pathway in PCSCs.

## Data Availability

The original contributions presented in this study are included in the article/Supplementary Material. Further inquiries can be directed to the corresponding authors.

## Supplementary Materials

The following supporting information can be downloaded at https://www.mdpi.com/article/10.3390/ph19010019/s1: Figure S1: Expression of LGR5 and CD133 in GD-treated ALDH+ HPAC cells; Figure S2: Purity of the stem cell population isolated from HPAC cells; Figure S3: Gedunin inhibits Shh in HPAC xenografts; Figure S4: Gedunin inhibits Shh in 5K PCSC xenografts.

## Abbreviations

The following abbreviations are used in this manuscript:

LGR5	Leucine-Rich Repeat-Containing G-Protein Coupled Receptor 5
CD133	Cluster of Differentiation 133
ALDH	Aldehyde dehydrogenase
GD	Gedunin
PDAC	Pancreatic ductal adenocarcinoma
CSC	Cancer stem cells
PCSC	Pancreatic cancer stem cells
LDA	Limited dilution assay
