# Supplementary material for: Gedunin Impacts Pancreatic Cancer Stem Cells Through the Sonic Hedgehog Signaling Pathway
Source: Pharmaceuticals (Basel). 2025 Dec 22;19(1):19. doi: 10.3390/ph19010019 (PMC12844995; doi:10.3390/ph19010019)
Supplement: Supplementary file 1 [file pharmaceuticals-19-00019-s001.zip › pharmaceuticals-4000232-supplementary.pdf]

Supplementary Material

Supplementary Figures

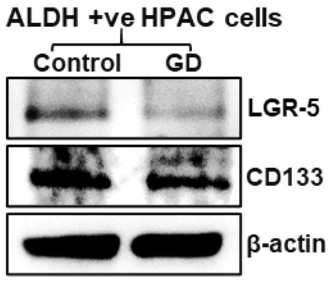

Figure S1. Expression of LGR5 and CD133 in GD-treated ALDH+ HPAC cells.

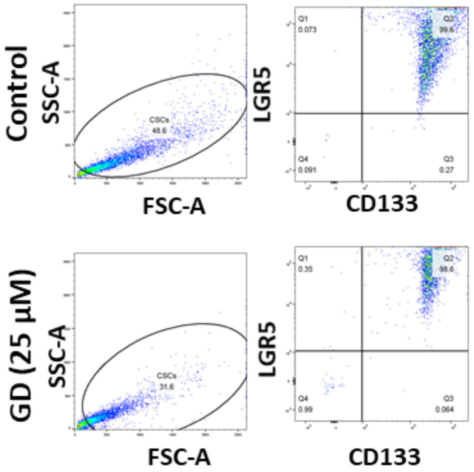

Figure S2. Purity of the isolated stem cell population from HPAC cells: PCSCs (LGR5+/CD133+) cells were isolated from HPAC cells after 24h of treatment with GD using EasySep Magnet cell sorter. Then, the cells were analyzed for purity using FACS.

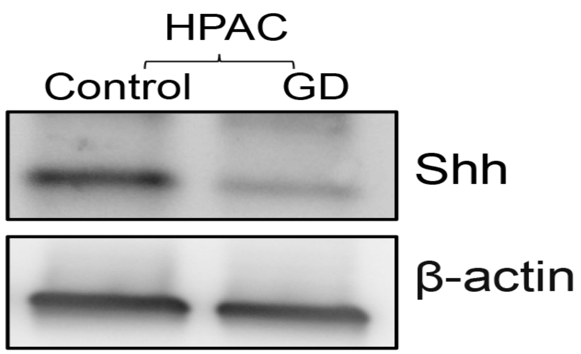

Figure S3. Gedunin inhibits the Shh in HPAC xenografts.

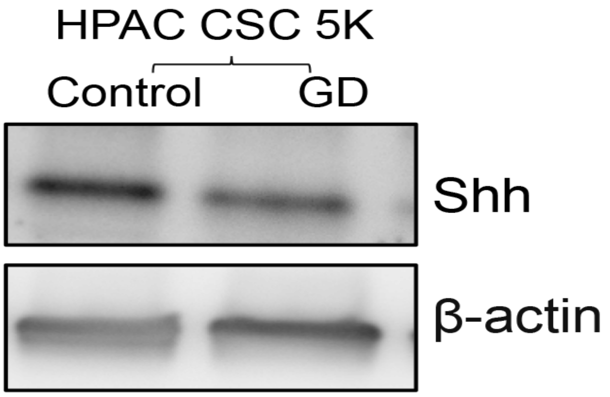

Figure S4. Gedunin inhibits the Shh in 5K PCSC xenografts.
